# Structural, Optical, and Electrical Parameters of Doped PVA/PVP Blend with TPAI or THAI Salt

**DOI:** 10.3390/polym15122661

**Published:** 2023-06-13

**Authors:** A. M. El-Naggar, Shadia Z. Brnawi, A. M. Kamal, A. A. Albassam, Zein K. Heiba, Mohamed Bakr Mohamed

**Affiliations:** 1 Research Chair of Exploitation of Renewable Energy Applications in Saudi Arabia, Physics & Astronomy Department, College of Science, King Saud University, Riyadh 11451, Saudi Arabia; amelnaggar@yahoo.com (A.M.E.-N.);; 2 Physics & Astronomy Department, College of Science, King Saud University, Riyadh 11451, Saudi Arabia; 3 Physics Department, Faculty of Science, Ain Shams University, Cairo 11566, Egypt

**Keywords:** PVA/PVP, TPAI, THAI, DC electric activation energy, optical

## Abstract

The 70% polyvinyl alcohol/30% polyvinyl pyrrolidone (PVA/PVP) polymer blends, with different weight ratios of tetrapropylammonium iodide (TPAI) or tetrahexylammonium iodide (THAI) salt, were prepared using dimethyl sulfoxide (DMSO) as a solvent. The X-ray diffraction technique was used to trace the crystalline nature of the formed blends. The SEM and EDS techniques were applied to figure out the morphology of the blends. The variation in the FTIR vibrational bands was used to investigate the chemical composition and the effect of different salt doping on the functional groups of the host blend. The influence of the salt type (TPAI or THAI) and its ratio on the linear and nonlinear optical parameters for the doped blends were investigated in detail. Absorbance and reflectance are highly enhanced in the UV region reaching a maximum for the blend with 24% TPAI or THAI; so, it can be employed as shielding materials for UVA and UVB types. The direct (5.1 eV) and indirect (4.8 eV) optical bandgaps were reduced continuously to (3.52, 3.63 eV) and (3.45, 3.51 eV) while increasing the content of TPAI or THAI, respectively. The blend doped with 24% wt TPAI exhibited the highest refractive index (around 3.5 in 400–800 nm). The DC conductivity is affected by the content and type of salt, its dispersion, and blend-salt interaction. The activation energies of different blends were obtained by applying the Arrhenius formula.

## 1. Introduction

A polymer blend is a mixture of two or more polymers that have been blended physically together to form a novel material with special physical characteristics. Polymer blending has gained attention as a simple and cost-effective method of producing polymeric materials with commercial applications [1,2]. Polyvinyl alcohol (PVA) has a semicrystalline nature, is nontoxic, has a high dielectric strength, and has a good charge storage capacity. It has a carbon chain backbone with hydroxyl groups (OH) attached to methane carbons. These OH groups are considered a source of hydrogen bonding and, therefore, assist in the creation of polymer composites [3]. Polyvinyl pyrrolidone (PVP) has an amorphous structure and a carbonyl group. In addition, its water solubility is very high, allowing it to be dispersed easily in a PVA polymer [4].

Organic salts such as tetramethylammonium iodide (TMAI), tetrapropylammonium iodide (TPAI), tetrabutylammonium iodide (TBAI), tetrahexylammonium iodide (THAI), pyridinium iodide, and picolinium iodide, which are non-volatile and stable under an atmospheric environment, were used in the fabrication of dye-sensitized solar cells (DSSCs) [5]. 

Loading polymers, or the blended polymers with suitable materials, can alter their properties. For example, the conductivity of PVA was enhanced as the amount of ammonium iodide increased [6]. Aziz et al. fabricated PVA-based gel polymer electrolytes doped with potassium iodide (KI) and TMAI salts for application in DSSCs [7]. The efficiency of the DSSCs was increased using gel polymer electrolytes based on a tetrahexylammonium iodide and MgI_2_ binary iodide system [8]. Doping a PVA/PVP blended polymer electrolyte with 40% TBAI showed the highest conductivity among the other TBAI doping ratios [9].

The TBAI organic iodide salt weight content in polyvinylidene fluoride-poly(methyl methacrylate)-ethylene carbonate polymer electrolytes influences the overall performance of dye-sensitized solar cells [10]. The ionic conductivity of PVA increased as the ammonium iodide salt increased up to 25 mol%, beyond which the conductivity reduced [5]. The electrolyte containing a 120% THAI and MgI_2_ salt mixture, with respect to the polyacrylonitrile (PAN) weight, displayed the maximum conductivity out of all samples measured with other doped ratios [11].

Ebnalwaled et al. developed novel UV shielding films based on PVA/Gelatin/0.01 CuO nanocomposite, where the nanocomposite can absorb different UV subtypes (UVA, UVB, and UVC [12]. Additionally, as the poly(methyl 2-methylpropenoate)polymeric films were doped with cyanopyridine, they were exhibited as UV and blue light filters [13]. Ti_3_C_2_T_x_ MXene/poly(vinyl alcohol) nanocomposite films effectively shield UVC and UVB types more than UVA types [14]. Lignin−PVA composite film showed great ultraviolet (UV) protective potential in both UVA and UVB regions [15]. UV light with a wavelength greater than 280 nm cannot be filtered by undoped poly(methyl methacrylate)/polystyrene thin films. Polymeric thin films’ UV-shielding efficiency can be improved by incorporating ZnO NPs at high concentrations [16].

The functional properties of PVA could be highly improved by blending with PVPs, such as permeability, hydrophilicity, elasticity, and stability in water, optically [17,18,19,20,21]. For preparing the PVA/PVP blends, DMSO is used as a solvent owing to its high dielectric constant and its role as a good plasticizer [22]. The MAPbI_3_ perovskite film prepared, in DMSO, displays the highest transmittance and the highest bandgap energy of the fabricated films [23].

In this study, the PVA/PVP polymer blend, loaded with different weight percentages of tetrapropylammonium iodide (TPAI) or tetrahexylammonium iodide (THAI) salt, was prepared using DMSO. The obtained composites were fully characterized by applying X-ray diffraction, FTIR, SEM, and EDS. The effect of different salts, TPAI or THAI, and their contents on the optical, linear, and nonlinear properties of the PVA/PVP blend were investigated using diffused UV reflectance techniques. The influence of different weight percentages on the conductivity of the blend was studied.

## 2. Methods and Materials

A 70% PVA/30% PVP (PVA/PVP) blend was formed using polyvinyl alcohol (PVA, MW = 50,000 g/mol, Acros Organics, 98%, Geel, Belgium) and polyvinyl pyrrolidone (PVP, MW = 40,000 g/mol, Alfa Aesar, Lancashire, United Kingdom). In total, 1.12 and 0.48 g of PVA and PVP, respectively, were dissolved separately in DMSO at RT using a magnetic stirrer until clear solutions were obtained. Then, the solutions were cast into Petri dishes and placed inside an electric oven at 70 °C for 4–5 days until the blends were created. The above procedures were repeated to form the 70% PVA/30% PVP/x wt % tetrapropylammonium iodide (TPAI, Sigma Aldrich, 98%, Taufkirchen, Germany) and 70% PVA/30% PVP/x wt % tetrahexylammonium iodide (THAI, Sigma Aldrich, 98%, Taufkirchen, Germany) blends. The weight ratio (x = 0, 6, 11, 16, 20, and 24) wt % of the TPAI or THAI salt to PVA/PVP polymer blend (weight) was determined from:

(1) $$X\left(\mathrm{wt}\;\%\right)=\frac{W_{f}}{W_{p}+W_{f}}\times 100$$

where *W_f_* and *W_p_* are the weights of the salts and blend, respectively.

The obtained blends have a thickness of 0.19–0.30 mm (measured using a digital micrometer). 

Data collection for the X-ray diffraction experiment was carried out with the assistance of a PANalytical diffractometer (X’pert MPD, Philips, Cu source, The Netherlands). Scanning electron microscopy (JED-2200 Series) and Fourier transform infrared (FTIR) spectroscopy were used to determine the morphology and vibration bands of the blends (Bruker Tensor 27 FTIR Spectrometer). For the EDS measurements, the samples were coated with gold. To perform the energy dispersive X-ray analysis (EDS), a 20 kV accelerating voltage, 120 s accumulation duration, and 6 mm window width were the variables used. The surface molar composition was determined using the ASA technique, ZAF correction, and Gaussian approximation. Using a spectrophotometer (JASCO–V-670), equipped with an integrating sphere assembly, the values for the diffused transmittance (*T*), absorbance (*A*), and reflectance (*R*) were gathered. The method described in references [24,25] was utilized in order to determine the *R* values prior to carrying out the calculations. The direct and indirect optical energy gaps’ values were obtained from the following equation [24]:

(2) $$\alpha hv=D{\left(hv-E_{g}\right)}^{r}$$

where *h*, *D*, *d*, and *α* (=2.303 × *A*/*d*) are Planck’s constant, incident light frequency, a constant defined as the disorder parameter, blended polymer thickness, and absorption coefficient, respectively. The value of *r* could be 0.5 or 2 for direct or indirect transition, respectively.

Using the equations found in Ref. [25], we were able to determine the refractive index (*n*), extinction coefficient (*k*), real (*ε_r_*) and imaginary (*ε_i_*) dielectric constant parts, linear optical susceptibility (*χ*^(1)^), nonlinear third order of the optical susceptibility (*χ*^(3)^) and nonlinear refractive index (*n*_2_) of the different blends.

DC conductivity (σ) measurements were performed at different temperatures using a setup consisting of an evacuated cryostat, operating with liquid nitrogen, a copper sample holder, a temperature controller, and a Keithley 6517-B electrometer.

The electrical conductivity (σ) is computed using the next formula:

(3) $$\sigma =\frac{d\times I}{V\times S}$$

where *d*, *S*, *I*, and *V* are the thickness of the sample, the cross-section area of the sample, electric current, and applied voltage, respectively.

## 3. Results and Discussion

### 3.1. Structural Investigation

**Figure 1:**
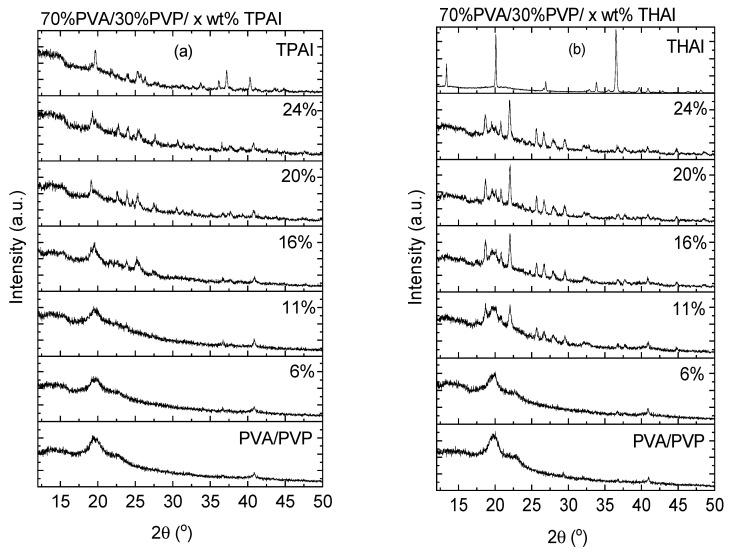
XRD diffraction data for the PVA/PVP blend doped with (**a**) TPAI or (**b**) THAI salt.

The X-ray diffraction data for the undoped and doped PVA/PVP blends, with TPAI or THAI salt, as well as the XRD data for TPAI and THAI salts are represented in Figure 1a and Figure 1b, respectively. The XRD data for the PVA/PVP blend exhibited a semicrystalline nature. As noticed from the graph, the diffraction patterns displayed a diffuse background with two broad diffraction peaks (halos) at roughly *2θ*~14.65° and 19.64°, which are shifted as the blend is loaded with TPAI or THAI, indicating the existence of a change in the chain packing of PVP. The presence of an amorphous phase with a low degree of crystallinity is indicated by a large amount of diffuse background and two broad peaks. PVA has two diffraction peaks at *2θ*~19.2° and 20.2° [26,27], whereas PVP has two broad humps at approximately 2*θ*~11.2° and 21.2° [28]. As the PVA blended with the PVP, the positions of the diffraction peaks are changed due to the formation of intermolecular hydrogen bonding between the PVA and PVP polymers [29]. The diffraction patterns of the doped blends have a similar trend as they are loaded with up to 11% TPAI or 6% THAI without any characteristic peaks from the TPAI or THAI salt, indicating a complete dissociation of the salt in the blend matrix. As the blend was doped with more TPAI or THAI salt, the broadness of the main peak decreased, the diffraction peaks of the different salts appeared, and its intensity rose as the percentage of TPAI or THAI increased, corresponding to the undissociated salt. This obviously shows the reduced, amorphous nature of the host blend. Additionally, the crystallinity of the doped blends increased as the blend was loaded with different ratios of different salts. The degree of crystallinity of the THAI-doped blends is greater than that of the TPAI-doped blends. 

**Figure 2:**
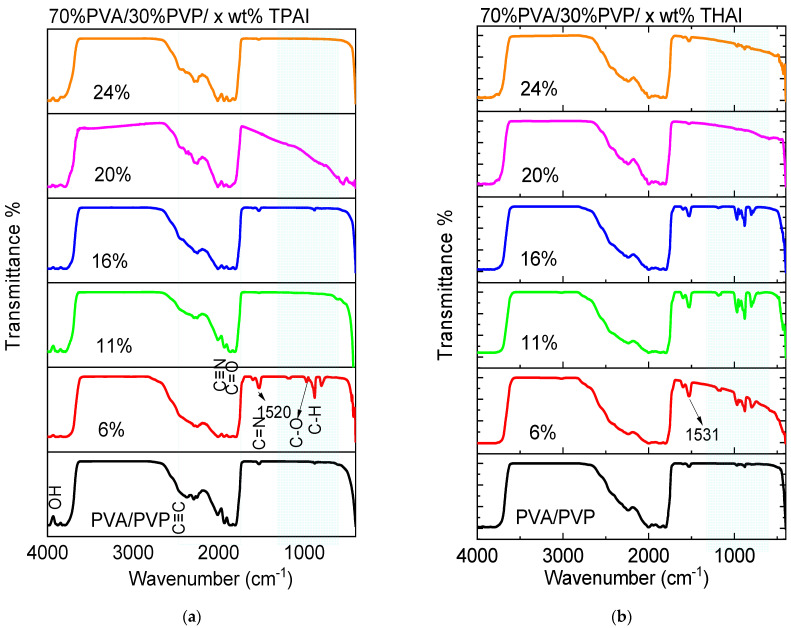
FTIR data for PVA/PVP blend doped with (**a**) TPAI or (**b**) THAI salt, the colors used to distinguish between the different samples.

The FT-IR absorption spectra (shown in Figure 2) in the range of 4000–400 cm^−1^ were recorded at room temperature for all samples. Pure and doped PVA/PVP blends with TPAI or THAI salt blends exhibit bands characteristic of stretching and bending vibrations in the spectra. The OH stretching band [30] describes the band at 3822 cm^−1^. The 2360 cm^−1^ band was identified as belonging to the C≡C bond [31]. For the PVA polymer, the C=O group is responsible for the vibration band at 1824 cm^−1^ [32]. The band appeared at 1998 cm^−1^, which is characteristic of C≡N bonds [30]. The blending and doping process significantly alters the intensity of the vibration bands in the wavenumber range of 2476–400 cm^−1^. PVA has C-O stretching at 950 cm^−1^ and C-H rocking bonds at 877 cm^−1^ [33]. The characteristic vibration of C=N (pyridine ring) is attributed to a small absorption band between 1520 and 1532 cm^−1^ [34]. The FT-IR spectra also show that there are band shifts and intensity changes compared to a pure blend. This shows that the salts have a profound impact on the host mixture.

**Figure 3:**
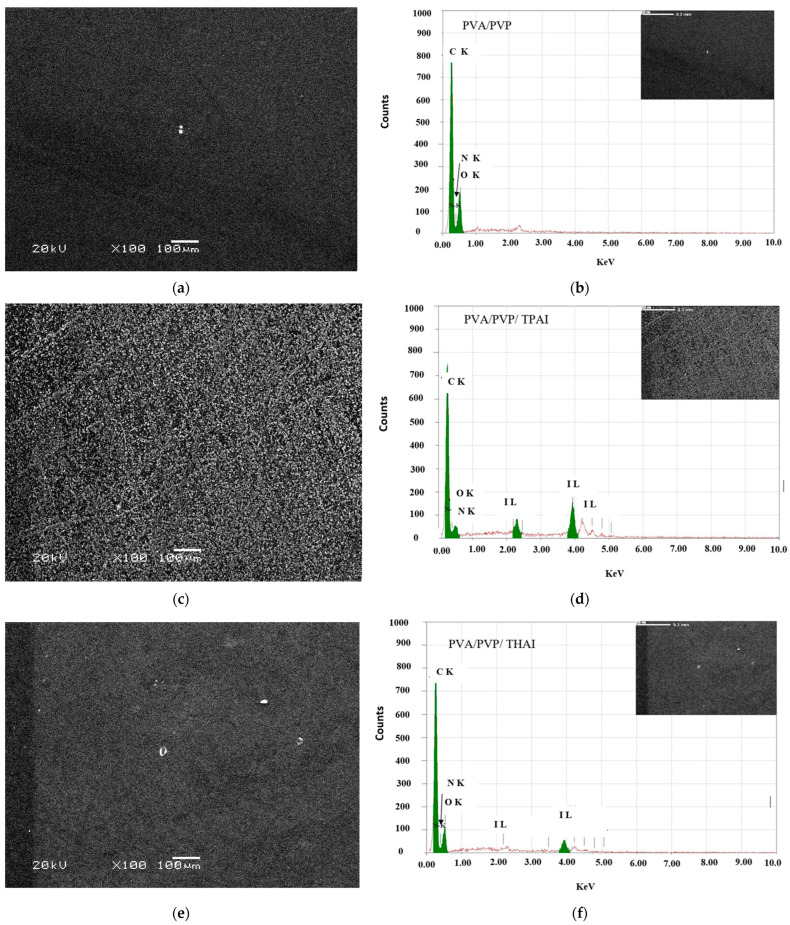
EDS and SEM images for (**a**,**b**) undoped and doped PVA/PVP blends with 16 wt % of (**c**,**b**) TPAI or (**e**,**f**) THAI salt.

The surface morphology of the obtained films was monitored by recording SEM images to observe the effect of loading the PVA/PVP polymer blend with TPAI or THAI salt. SEM images of unloaded and TPAI/THAI-loaded polymer blends are shown in Figure 3a,c,e. For the pure polymer blend, some bright spots could be seen on the surface’s homogeneous smoothness. These white patches show some immiscibility in the blend. After loading, the surface became slightly rougher and some white patches developed; the amounts of these patches are greater in the doped blend with THAI than in the blend with TPAI salt. In addition, the blend with TPAI is rougher than the blend with THAI, which will affect the different properties of the blends. Similar results were observed upon loading the PVA/PVP with lithium bromide [32]. This change may be confirmed by the different interactions and complexations between the different salts and the host blend. All blends contain C, N, and O, but doped blends also contain iodine (I), according to the corresponding EDS analysis (Figure 3b,d,e).

### 3.2. UV Spectra Analysis

**Figure 4:**
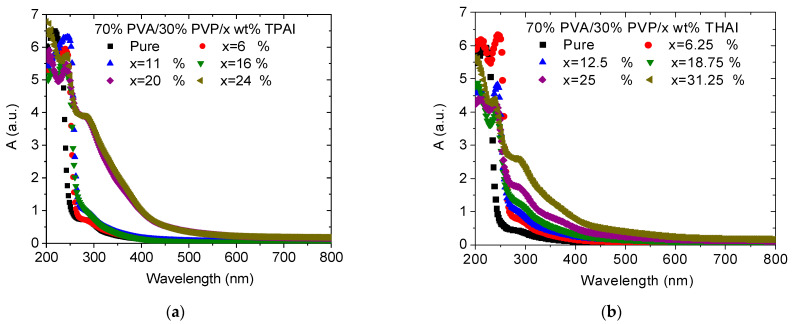

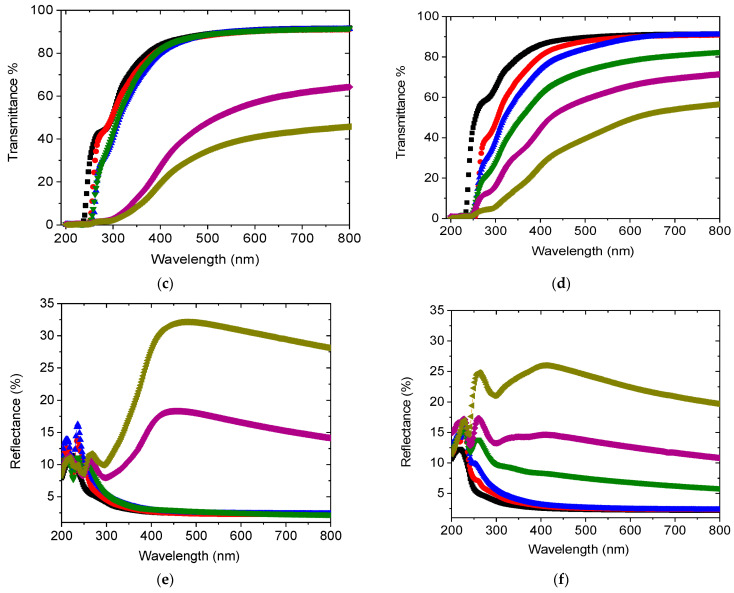
Wavelength dependent on the (**a**,**b**) absorbance, (**c**,**d**) transmittance, and (**e**,**f**) reflectance for PVA/PVP/x wt % TPAI or THAI blends.

The optical characteristics of any material are strongly affected by the interactions between the incident light waves and the material. Figure 4 displays the wavelength-dependent absorbance (*A*), transmittance (*T*), and reflectance (*R*) data for pure and doped blends with TPAI or THAI salt. A large absorbance spectrum is observed in the UV range, while a small absorbance is detected in the visible range. The absorbance spectra of all blends have bands at 217 and 252 nm, which are associated with n → π* and π → π* electronic transitions, respectively [35]. As the blend was doped with TPAI, the absorbance changed irregularly. Whereas the amount of TPAI increased up to 16%, the absorbance increased slightly, then increased largely beyond this doping range. Furthermore, as the blend was doped with THAI, the absorbance increased regularly as the amount of THAI increased. This increase is caused by the agglomeration of TPAI or THAI at the surface of the blend. In addition, this increase illustrates the variation in the type of interaction between the molecules of PVA/PVP and different salts. The absorbance of the doped blend with 24 wt % TPAI salt is higher than that of the THAI salt. The increase in the absorbance spectra of the doped blend, as compared with the pure blend in the wavelength range of 290–420 nm, nominated the doped blends to be used as shielding materials for the UVA and UVB types. All these modifications in the absorbance of the blend indicated the occurrence of crystallinity modifications, bandgap structure, and the refractive index of the host blend upon doping with different amounts of salts [35]. The transmittance of the PVA/PVP blend displayed the highest value, ~90%, and it decreased irregularly or regularly as it was doped with the TPAI or THAI salt, respectively. The doped blend with 24 wt % TPAI has the lowest transmittance value as compared with the other doped blends with other ratios, whether it is doped with TPAI or THAI salt. The scattering phenomenon is considered the most significant factor that affects the optical transmittance data of the doped blends under investigation. The most common possibilities for scattering are surface and grain boundary scattering, which are present due to the existence of novel defects or disorders within the blend matrix after doping. The most common scattering processes responsible for the reduction of the transmittance spectra are surface and grain boundary scattering defects, as well as the degree of crystallinity of the blend [36]. All the blends exhibited irregular or regular redshifts as the amount of TPAI or THAI salt doping increased in the blend, respectively. This shift demonstrated that the decrease in the optical bandgap of the blends was due to the formation of sub-levels between the highest occupied molecular orbital (HOMO) and the lowest unoccupied molecular orbital (LUMO) states inside the gap of the host blend matrix. Additionally, doping the PVA/PVP blend with TPAI or THAI salt increased its reflection. The blend doped with 24 wt % TPAI has a higher reflectance than the corresponding blend with THAI salt.

**Figure 5:**
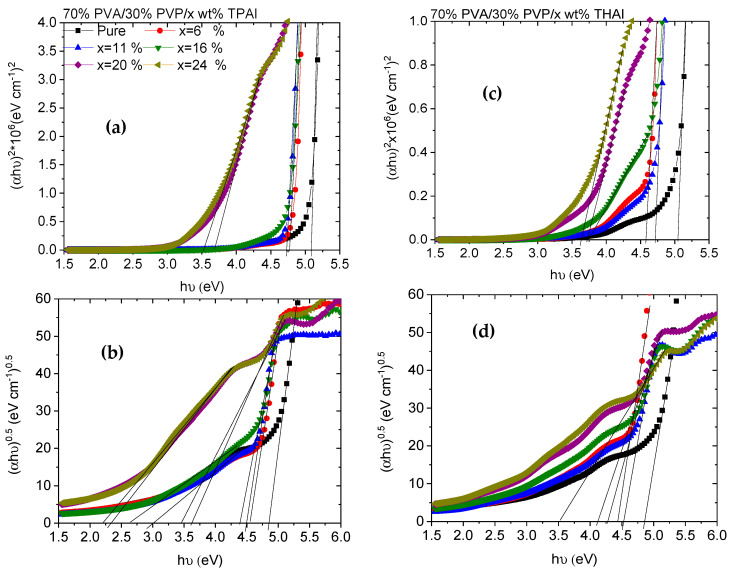
(**a**,**c**) Direct and (**b**,**d**) indirect Tauc relations for PVA/PVP/x wt % TPAI or THAI blends, respectively.

The direct and indirect optical bandgap (*E_g_*) values of different blends can be computed from the relation between (*αhν*)^2^ and (*αhν*)^1/2^ vs. *hν* (Figure 5) by extending the straight portions of the plots to the intercept (*hν*) axis. The direct and indirect *E_g_* values for all blends are listed in Table 1. According to the table, the direct and indirect *E_g_* values of PVA/PVP are 5.1 and 4.86 eV. These values were reduced as the amount of TPAI or THAI was increased: 3.52, 3.45, 2.21, or 3.63, 3.51 eV, respectively. The reduction in the energy gap values indicated the induced variations within the band gap structure in the form of novel localized energy states within this forbidden region, due to the increase in the number of defects and the change in the degree of disorder resulting from the doping (SEM, Figure 3). This reduction was detected previously as PVA doped with NH_4_I. This reduction is due to the inter- and intra-molecular hydrogen bonds created between ammonium ions and adjoining hydroxyl groups [37].

**Figure 6:**
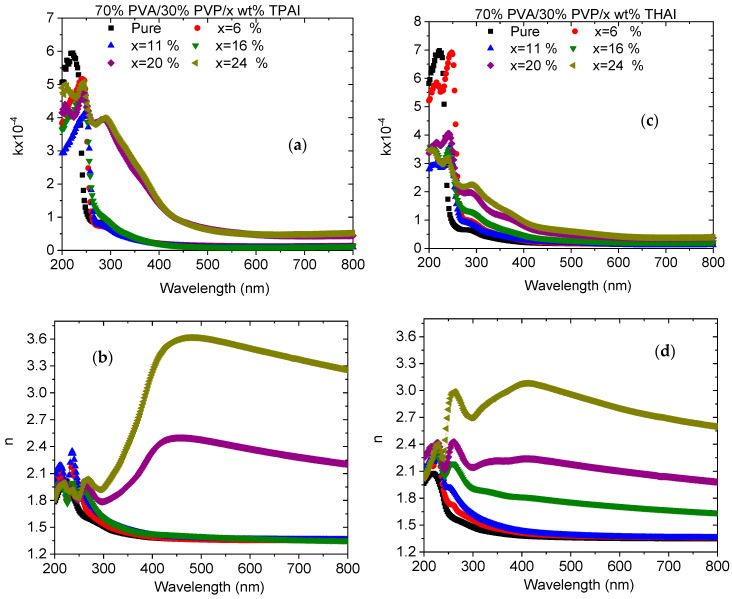
(**a**,**c**) Extinction coefficient and (**b**,**d**) refractive index for PVA/PVP/x wt % TPAI or THAI blends, respectively.

The extinction coefficient (*k*) plays a vital role in the determination of numerous optical parameters; particularly those connected with the absorption of light waves in the medium and the dielectric constants. The *k* value refers to the fraction of light lost owing to the scattering and absorption per unit distance of the penetration medium. The *k* values for all pure and doped blends with TPAI or THAI, respectively, are represented in Figure 6a,c as a function of the wavelength of the incident photon. The graph reveals that the *k* value first increased and then decreased as the wavelength increased. In the shorter wavelength range, the *k* value reduced as PVA/PVP loaded with different ratios of the different salts. Beyond this range, the *k* value increased as the PVA/PVP blend was loaded with different amounts of TPAI or THAI salt. The *k* values of the blend doped with 24 wt % TPAI are higher than the corresponding blend loaded with THAI. This indicated that the intensity of the light is attenuated further by scattering and absorption in the blend doped with TPAI than in the blend doped with THAI. The increase in k values was associated with an increase in the number of defects or disorders in the host blend after doping with various amounts of salts.

The refractive index (*n*) is an essential optical parameter that is needed to construct the optical-electronic components that are used in many applications, such as interference filters, modulators, and waveguides. The wavelength dependence of the *n* values for all blends is shown in Figure 6b,d. The increase in the *n* values, upon doping the PVA/PVP blend with TPAI or THAI, maintains the induced variations within the blend structure. Beyond *λ* > 300 nm, all blends exhibited a normal dispersion, except blends doped with higher amounts of TPAI or THAI, which exhibited anomalous and normal dispersions. This modification may be caused by the modulation in the structure of the host blend matrix due to the bonding arrangement and the dispersion of TPAI or THAI over the blend matrix [38]. The increase in the *n* value is due to the increase in the number of defects (disorders) caused by doping. Moreover, the doped blend with 24 wt % TPAI exhibited the highest *n* value among all the studied blends. The reduction in the *n* values with *λ* for all blends at higher *λ* ranges can be explained as follows, at low-energy incident photons, the degree of the polarizability of the polymer molecules decreased as the *n* value decreased. 

**Figure 7:**
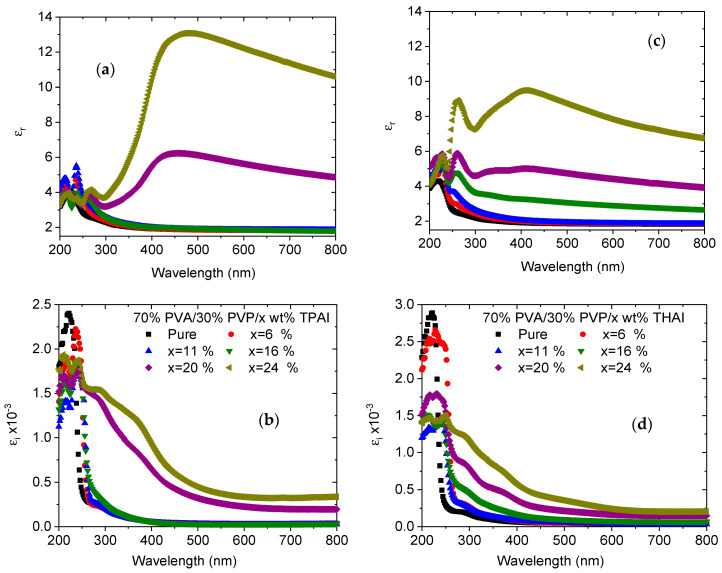
(**a**,**c**) Real and (**b**,**d**) imaginary dielectric parts for PVA/PVP/x wt % TPAI or THAI blends, respectively.

The real part of the dielectric constant (*ε_r_*) is associated with the dispersion of the electromagnetic waves when they move throughout the material, and it is also responsible for the reduction in the propagation speed of the waves throughout the blend. Furthermore, the imaginary part (*ε_i_*) represents the energy absorption from the electric field due to dipole movement; consequently, it gives a measure of the disruptive rate of the wave in the blend [39]. The wavelength dependence of the *ε_r_* and *ε_i_* values of all blends is shown in Figure 7. In each blend, the values of *ε_r_* are greater than the value of the corresponding *ε_i_*_._ This is because the values of *ε_r_* are strongly correlated with the *n* value, whereas the values of *ε_i_* are based on *k* values. In the visible range, the *ε_r_* and *ε_i_* values increased as the amount of TPAI or THAI increased in the PVA/PVP blend. The highest *ε_r_* and *ε_i_* values were obtained as the PVA/PVP blend was doped with 24% TPAI. The changes in the dielectric constants with the incident photon energy demonstrated that there are some interactions that take place between the incident photons and the free electrons in the studied range. 

**Figure 8:**
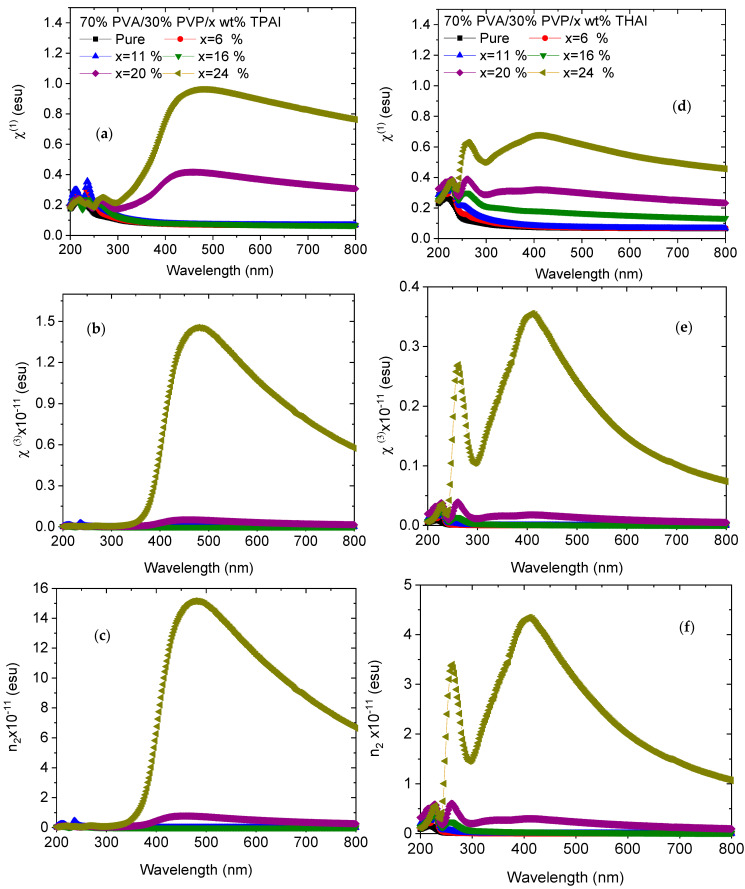
Wavelength dependent of (**a**,**d**) χ^(1)^, (**b**,**e**) χ^(3)^, and (**c**,**f**) n_2_ for PVA/PVP/x wt % TPAI or THAI blends, respectively.

The linear optical susceptibility (*χ*^(1)^) of the material is one of the optical parameters that can be employed to compute the third-order nonlinear optical susceptibility (*χ*^(3)^). *χ*^(3)^ is a complex optical parameter that points to the nonlinearity trend of the material. The nonlinear refractive index (*n*_2_) is a significant parameter in the formation of optical systems and high-power laser sources. The wavelength-dependent nonlinear optical parameters (NLO = *χ*^(1)^, *χ*^(3)^, and *n*_2_) for all blends are displayed in Figure 8. It was noticed that the NLO parameters had a similar attitude. The NLO parameters were increased as the PVA/PVP blend was doped with different ratios of TPAI or THAI salt. In the UV range, the NLO parameters of doped blends with THAI are higher than those of the corresponding blends doped with TPAI. The situation is reversed in the visible range. The improvement in the NLO parameters of doped blends may be caused by the modification in the electronic structure of the host blend upon doping with TPAI or THAI. Doped blends with improved NLO parameters may be suitable for use in a variety of nonlinear optical potential applications.

### 3.3. DC Electrical Conductivity

**Figure 9:**
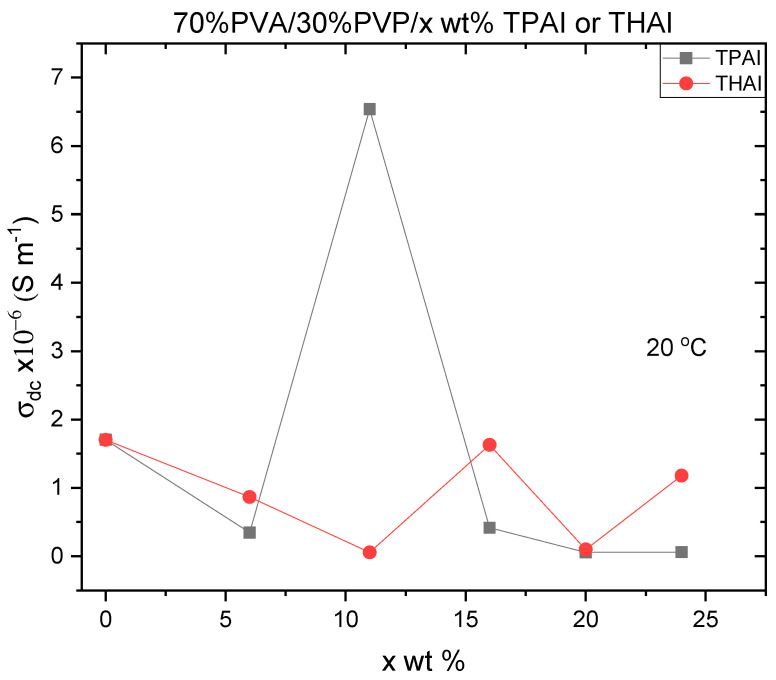
The DC conductivity for PVA/PVP/x wt % TPAI or THAI blends at room temperature.

Figure 9 reveals the variation of the DC conductivity (*σ_dc_*) with TPAI or THAI concentration in the blends at room temperature. The conductivities of all blends are relatively low (10^−6^–10^−8^ S m^−1^). The conductivity exhibited the highest value for the PVA/PVP blend loaded with 11 wt % TPAI or 16 wt % THAI (percolation ratio). A similar result was obtained as poly(ethylene oxide) (PEO)–LiClO_4_ dopped Al_2_O_3_, where ionic conductivity passes a maximum value at 12 wt % Al_2_O_3_, then reduced with further doping. The authors argued the change in the conductivity was due to the change in the crystallinity of the host polymer as a result of the random distribution of fine Al_2_O_3_ powder. This doping introduces the topological disorder to the polymer electrolyte. A polymer chain is more mobile in its individual segments when it is in the amorphous phase or in the less ordered regions. Ionic conductivity drops above 12 wt % Al_2_O_3_ because inorganic fillers have a tendency to act as an insulator or an agglomerator, slowing down the movement of ions [40]. Pan et al. studied the effect of electron concentration on electrical conductivity in in situ Al-TiB_2_ nanocomposites. Defects such as interfaces and dislocations are produced and the preferred growth modes of Al grains are altered by the presence of TiB_2_ nanoparticles [41]. By increasing the defect density of states, these defects can both increase the number of free electrons and the strength of the electron lattice’s effects. In addition, the Fermi velocity and density of states exhibit mismatches and discontinuities at these interfaces. Both of these effects play a role in the scattering of free electrons and the formation of bound electron states and, hence, affect electrical performance [41,42].

The variation in DC conductivity, in our case, can be attributed to changes in the number of mobile charge carriers, as well as variations in the crystallinity nature of the blend, which affects the energy barrier and, thus, ion transport. Whereas the decrease in DC conductivity (*σ_dc_*) may be due to undissociated salt causing ion aggregation, resulting in the formation of ion clusters and stronger ion-ion interactions, which may impede the polymer’s backbone segmental motion and, thus, ultimately cause a decrease in conductivity. Similar results were observed with PVA doped with NH_4_I [6].

**Figure 10:**
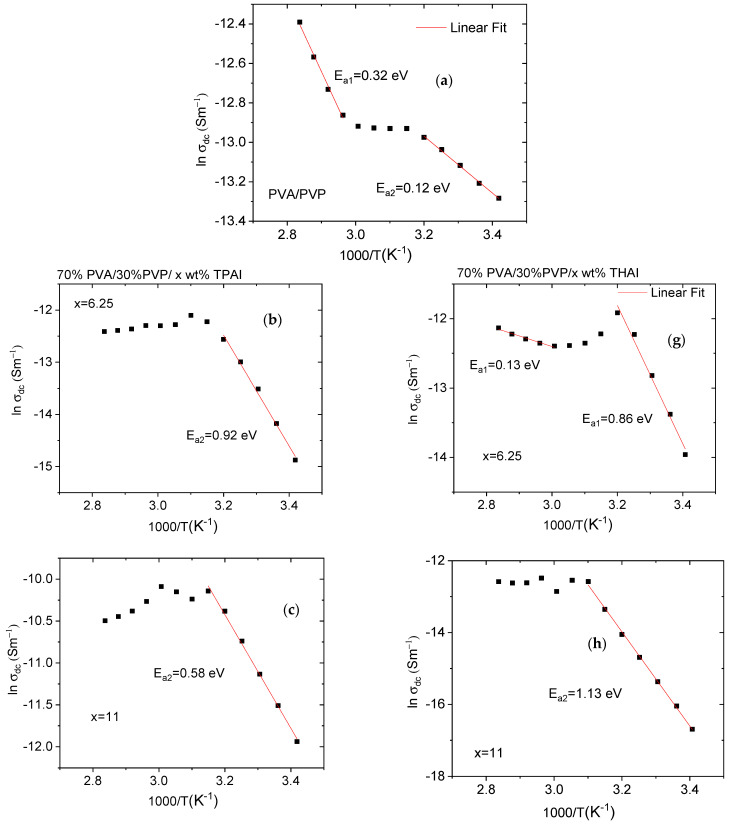

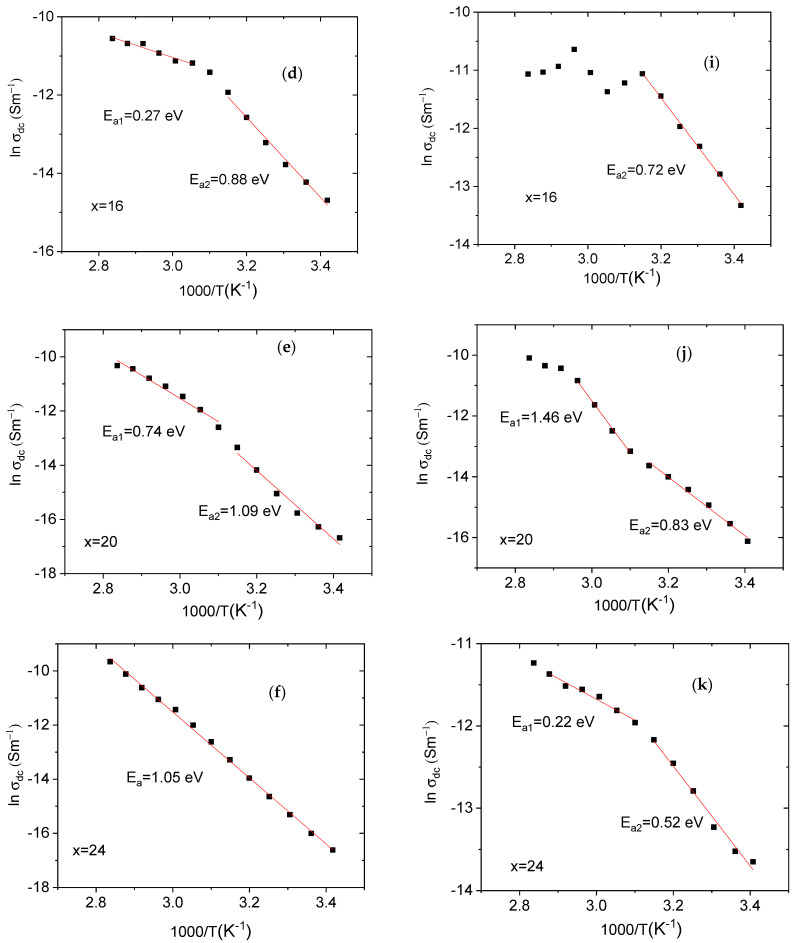
lnσ as a function of temperature for (**a**) pure PVA/PVP and doped blends with (**b**–**f**) TPAI or (**g**–**k**) THAI salts.

Figure 10 displays the variation of ln (*σ_dc_*) with the reciprocal of temperature (1000/*T*) for all blends. The DC conductivity for all blends can be illustrated by the Arrhenius formula [43]:

(4) $$\sigma_{dc}=C\;\exp(-E_{a}/k_{B}T)$$

where *E_a_*, *C*, and *k_B_* are the DC conductivity activation energy, the temperature-independent constant (which depends on the physical and chemical features of the blends), and Boltzmann’s constant, respectively.

As revealed from the graphs, the DC conductivity is affected by the amounts and types of salt, dispersion, and blend-salt interaction [43]. The values of *E_a_* can be obtained using the least square fitting of the last equation for all curves for all blends. It is worth noting that pure and doped PVA/PVP, with various salts (types and ratios), have two activation energies (*E_a_*_1_ and *E_a_*_2_). The *E_a_*_1_ and *E_a_*_2_ values of the PVA/PVP blend are (0.32, 0.12) or (0.30, 0.59) eV for the TPAI or THAI, respectively. These values changed irregularly depending on the type of salt and its amount in the blend (Table 2). The variations in the *E_a_* values may be due to the variation in the number of delocalized charge carriers formed in each blend [44]. 

## 4. Conclusions

PVA/PVP blends loaded with x wt % TPAI or THAI were produced. The XRD patterns manifested only an amorphous phase with humps, resembling PVA/PVP, up to x = 11% TPAI and 6% THAI. For higher contents of salts, XDR patterns disclosed crystalline peaks corresponding to TPAI or THAI. The PVA/PVP blend absorbance changed a little upon loading with TPAI up to 16%, but largely increased, in the range 280–500 nm, for x ≥ 24%. For THAI, the absorbance increased regularly with the salt percent. For composites with loading x ≥ 24%, the reflectance and refractive index are highly enhanced. The direct and indirect optical bandgaps were reduced continuously with a percentage of TPAI or THAI. Beyond *λ* > 300 nm, all blends exhibited a normal dispersion, except those with x ≥ 24% TPAI or THAI, which exhibited anomalous and normal dispersions. In the visible range, the dielectric constant values increased as the percent of TPAI or THAI increased. In the UV range, the NLO parameters of doped blends with THAI are higher than those of the corresponding blends doped with TPAI. The situation is reversed in the visible range. The nonlinear optical parameters (*χ*^(1)^, *χ*^(3)^, and *n*_2_) are greatly enhanced, by a factor of ten times, for the composites with 24% TPAI or THAI, nominating them for use in a variety of nonlinear optical potential applications. The conductivities of all blends are relatively low. The conductivity exhibited the highest value for the PVA/PVP blend loaded with 11 wt % TPAI or 16 wt % THAI. The activation energies changed irregularly depending on the type of salt and its percentage in the blend.

## Figures and Tables

**Table 1:** The direct and indirect optical bandgaps for PVA/PVP and PVA/PVP/x wt % TPAI or THAI blends.

	*E_g_* (eV)				
wt % TPAI	Direct	Indirect	wt % THAI	Direct	Indirect
Pure	5.1	4.86	Pure	5.03	4.86
x = 6%	4.78	4.56	x = 6%	4.72	4.53
x = 11%	4.74	4.49, 3.01	x = 11%	4.59	4.44
x = 16%	4.72	4.39, 2.62	x = 16%	3.77	4.29
x = 20%	3.68	3.62, 2.32	x = 20%	3.73	4.12
x = 24%	3.52	3.45, 2.21	x = 24%	3.63	3.51

**Table 2:** The activation energies for PVA/PVP and PVA/PVP/x wt % TPAI or THAI blends.

wt % TPAI	*E*_*a*1_ (eV)	*E*_*a*2_ (eV)	wt % THAI	*E*_*a*1_ (eV)	*E*_*a*2_ (eV)
Pure	0.32	0.12	Pure	0.30	0.59
x = 6%	---	0.92	x = 6%	0.13	0.86
x = 11%	---	0.58	x = 11%	---	1.13
x = 16%	0.27	0.88	x = 16%	---	0.72
x = 20%	0.74	1.09	x = 20%	1.46	0.83
x = 24%	1.05	1.05	x = 24%	0.22	0.52

## Data Availability

The authors confirm that the data supporting the findings of this study are available within the article.
